# Dietary Folate Deficiency Promotes Lactate Metabolic Disorders to Sensitize Lung Cancer Metastasis through MTOR-Signaling-Mediated Druggable Oncotargets

**DOI:** 10.3390/nu15061514

**Published:** 2023-03-21

**Authors:** Wan-Jing Chen, Su-Yu Huang, Yi-Wen Chen, Yi-Fang Liu, Rwei-Fen S. Huang

**Affiliations:** 1 Program in Nutrition and Food Science, Fu Jen Catholic University, New Taipei City 242, Taiwan; 2 Department of Nutritional Science, Fu Jen Catholic University, New Taipei City 242, Taiwan

**Keywords:** dietary folate deficiency, lactate metabolic disorders, lung cancer, metastasis, mTORC1 signaling, oncotargets

## Abstract

Lactate metabolism plays a pivotal role in cancers but is often overlooked in lung cancer (LC). Folate deficiency has been linked to lung cancer development, but its impact on lactate metabolism and cancer malignancy is unclear. To investigate this, mice were fed either a folate-deficient (FD) or control diet and intrapleurally implanted with lung cancer cells pre-exposed to FD growth medium. Results showed that FD promoted lactate over-production and the formation of tumor oncospheroids (LCSs) with increased metastatic, migration, and invasion potential. Mice implanted with these cells and fed an FD diet developed hyperlactatemia in blood and lungs. This coincided with increased expression of hexokinase 2 (HK2), lactate dehydrogenase (LDH), and decreased expression of pyruvate dehydrogenase (PDH). Pre-treatment of the FD-LCS-implanted mice with the mTORC1 inhibitor, rapamycin, and the anti-metabolic drug metformin abolished FD/LCS-activated mTORC1 and its targets including HIF1α, HK2, LDH, and monocarboxylate transporters (MCT1 and MCT4), which coincided with the reduction in lactate disorders and prevention of LC metastasis. The findings suggest that dietary FD promotes lactate metabolic disorders that sensitize lung cancer metastasis through mTOR-signaling-mediated targets.

## 1. Introduction

As an essential nutrient and food functional component, the human body demands sufficient dietary folate for normal one-carbon metabolism in de novo nucleotides synthesis, bioenergetics, and redox balance to support cellular proliferation and organism growth [1]. Dietary folate deficiency (FD) results in a folate-deficient vascular and tissue microenvironment which stresses cellular one-carbon metabolism towards oncogenic transformation with increased risks of cancer malignancy development [2,3,4]. Depending on the magnitude of tissue folate depletion, folate deprivation of cancer cells leads to cell cycle arrest and apoptotic cell death [5,6], and/or selected immortal cancer cells with a stemness phenotype of enhanced cancer metastatic potential in invasion, migration, and self-renewal capability to adapt anchorage-independent growth in the vascular tumor microenvironment (TME) [7,8,9,10,11]. Numerous studies have also shown that FD-diet-fed animals developed systematic bioenergetic deficits due to dysfunctional mitochondria (mt) oxidative phosphorylation (OXPHOS) and elevated mt oxidative damage in DNA, lipids, and proteins of peripheral tissues [12,13,14]. It is as yet not completely understood how cancer cells may survive the FD-induced bioenergetics crisis to establish their metastatic behavior which demands high energy support.

A recent advance has proposed that reprogramming the lactate metabolism in the TME of the host regulates tumor progression and metastasis [15]. When primary cancer cells migrate from hypoxic tumor bulk to the vascular avenue for distant metastasis, they demand a high energy supply for anchorage-independent growth survival from the scarce-nutrient TME. Even under a normoxic TME, cancers with dysfunctional mt OXPHOS metabolize most glucose carbon to lactate via anaerobic glycolysis as the alternative fuel for proliferation, known as the Warburg effect [16]. Lactate over-production in tumor-adjacent tissues triggers metabolic-stress-signaling lactate mobilization to acidify the TME and to sustain the cancers’ fuel demand for metastatic development. Such a metabolic coupling event in tumors/tumor-adjacent tissues was named the reverse Warburg effect to support metabolic symbiosis of fuel lactate utilization in malignant cancers [17,18]. Clinical studies have indicated hyperlactatemia is the key predictor of cancer metastases, tumor recurrence, and cancer-related mortality [19,20,21]. Blockage of the reverse Warburg effect to remodel cancer lactate metabolism has been proposed to be a promising therapeutic approach for treating cancer malignancy [22,23].

Lung cancer is the leading cause of cancer-related mortality worldwide [24]. Early stage lung cancers (LCs) are potentially curable, whereas many patients develop metastatic diseases with poor prognostic outcomes. A recent advance in genomic and metabolomic studies suggests that lactate is the distinctive oncometabolite signature of human LC with aggressive oncological behavior [25]. The environmental factors to reprogram lactate metabolism in LC, however, remain poorly defined. Insufficient dietary folate intake has been associated with increased risks of LC carcinogenesis among former and current smokers [26,27]. Several studies have examined an association between circulating folate status and LC carcinogenesis, but results were inconclusive [28,29]. Despite the mixed outcomes in the previous findings, none has explored whether insufficient folate in the diet exerts a metabolic impact on tissue lactate disorders and advanced lung malignancy. There is currently no evidence to unveil whether and the working mechanisms by which such a dietary folate intake factor may regulate lactate metabolism to drive metastatic LC progression. We, therefore, established the in vitro FD-exposed lung carcinoma cell and dietary FD intervention of an FD-exposed LC-implanted mouse model to evaluate (1) the causal effects of dietary FD exposure on lactate metabolic disorders in the TME and LC metastasis and (2) to depict the lactate metabolic effects of dietary FD on the metabolic stress regulator, the mTOR-signaling pathway, and its druggable oncotargets in relation to LC metastasis. The clinical relevance and application are then discussed.

## 2. Materials and Methods

### 2.1. Chemicals and Reagents

Folic acid (FA, pteroylmonoglutamic acid), metformin (met), and rapamycin (Rap) were obtained from Sigma Chemical Co. (St Louis, MO, USA). RPMI 1640 medium was purchased from Invitrogen (Grand Island, NY, USA). Penicillin, streptomycin, trypsin, and fetal bovine serum were from Gibco Laboratories (Grand Island, NY, USA). Matrigel was purchased from BD (Franklin Lakes, NJ, USA). mTORC1 inhibitor rapamycin (Rap) and antibodies of Sox2, ALDH1A1, E-cadherin, vimentin, mTORC1, HIF1α, HK2, LDHA, VDAC, G6PDH, PDHE1, ACLY, GLUT1/4, and MCT1/4 were from Cell Signaling Technology (Boston, MA, USA). Antibodies for beta-actin and GAPDH were from Millipore (Temecula, CA, USA). Millicell culture inserts containing a thin film polycarbonate (PCF) membrane were obtained from Millipore (Billerica, MA, USA). A folate-deficient, L-amino-acid-defined diet was specially formulated by Teklad Global Diets^®^ (Madison, WI, USA). *Lacticaseibacillus casei (L. casei)* (BCRC^®^10697) was purchased from the Bioresource Collection and Research Center (Taipei, Taiwan).

### 2.2. Cell Culture and Treatment

The Lewis lung carcinoma cells (LC) were obtained from the National Development Center of Biotechnology (Taipei, Taiwan). LC cells were maintained as a monolayer culture in a control RPMI 1640 medium with 2 uM folic acid and supplemented with 2.0 g/L of sodium bicarbonate, 10% (*v*/*v*) fetal bovine serum (FBS), 100 U/mL of penicillin, and 100 mg/mL of streptomycin at 37 °C in a humidified 5% CO_2_ incubator. Folate-deficient (FD) cells were cultured in folate-deficient RPMI 1640 medium supplemented with folic acid at final concentration of 10 nM to mimic serum folate levels of cancer patients in marginal folate deficiency [3,4].

### 2.3. In Vitro Transwell Invasion Assay

Matrigel (BD Biosciences, Franklin Lakes, NJ, USA) was coated onto transwell membranes (Corning Costar, MA, USA) for 12 h. After 72 h of control/folate deprivation, cells (1 × 10^4^) were resuspended in serum-free medium, then seeded into the upper chamber, and 0.6 mL medium with 10% FBS was added to the lower chamber as a chemoattractant. After 24 h incubation, a cotton swab was used to remove non-invading cells on the upper surface of the membrane. The invasive cells, which crossed to the lower surface, were fixed with 4% paraformaldehyde and stained with 0.1% crystal violet (Merck, Darmstadt, Germany). The number of invading cells were counted from 9 randomly selected visual fields with an inverted microscope at 200× magnification. Data were obtained from three independent experiments.

### 2.4. Wound Healing Assay

LC cells were seeded onto 12-well plates and incubated with control medium (2 µmol folate) or folate deprivation medium (10 nmol folate) for 48 h. When cell confluence reached approximately 80%, a 10 μL pipette tip was used to create wounds, which were made by scraping the cell layer across each culture plate. After wounding, debris was removed by washing the cells with PBS. Wounded cultures were incubated in serum-free medium for 48 h, and then three fields (10×) were randomly taken from each scratch wound and visualized by microscopy to assess cell migration. The migration area of the scratched-wound image was analyzed at 0, 24, and 48 h using Image J software, Version 1.54b (National Institutes of Health, New York, NY, USA).

### 2.5. Spheroid Formation Assay

Oncosphere formation was induced by seeding the control and FD cells (1000 cells/mL) in the low-attachment 6-well plate in suspension tumorsphere medium which comprised serum-free Dulbecco’s modified Eagle’s medium supplemented with 2% B-27^®^ (Life Technologies, Carlsbad, CA, USA) plus 20 ng/mL human recombinant epidermal growth factor, 10 ng/mL fibroblast growth factor, 100 IU/mL penicillin, and 100 μg/mL streptomycin. The oncospheres (tight, spherical, non-adherent masses >40 um in diameter) in each well were photographed and counted under a phase-contrast microscope at 200× magnification.

### 2.6. Animal Study and Drug Treatment

The experimental protocols were approved by the Institutional Animal Care Committee of Fu Jen Catholic University (approval number: A10260) in accordance with the National Institute of Health guide for the care and use of laboratory animals. Male C57BL/6 mice at 6 weeks of age were fed an amino-acid-defined folate-deficient diet to FD group or a control diet to control group (with folic acid at 2 mg/kg of diet) for 14 days, which were made by Teklad Global Diets^®^ (Supplementary Table S1). Then, the FD and control mice were intrapleurally implanted with saline (sham group) and in vitro control or FD-exposed lung cancers (1 × 10^6^ cells) at various tumor malignancy stages. Drug treatment of rapamycin (Rap: 0.21 mM) and metformin (Met: 1.5 mM) at dose of 200 mg/mL/day was conducted by intraperitoneal injection to the control and FD mice one week prior to LCS transplantation. After 14 days, the mice were sacrificed for tumor burden, multiplicity, weight, and size evaluation. Blood samples and tissues were collected for fuel status analysis. Paired lung tumor tissues were removed and fixed in 10% neutral buffered formalin solution for histological analysis.

### 2.7. Western Blotting

Total protein was extracted and Western blotting on total and phosphorus–protein expression was performed according to a previously described protocol [7]. In brief, the quantified proteins were resolved on SDS–polyacrylamide gels and transferred onto a polyvinylidene difluoride membrane. The membranes were then immunoblotted with specific primary antibodies against beta-actin, GAPDH, metabolic enzymes (HK2, LDHA, VDAC, PDH, ACLY, and G6PDH), and components of mTOR-signaling pathways (Akt, mTOR, HIF1α, S6K, AMPK, and IRS-1) and were then incubated with the corresponding horseradish-peroxidase-conjugated IgG secondary antibody. All immunoblots were visualized using a WesternBright ECL substrate kit (Thermo, MA, USA). Actin and GAPDH were used as a loading control.

We performed Western blot normalization on the Image Lab^TM^ software tools. Background-subtracted protein signals were quantified for both the target protein and the loading control in each lane of the blot. Then, we selected a reference lane and determined a normalization factor by separating the signal from the reference lane to arrive at a normalization factor for each lane. All calculations were performed by the software, including the normalization factor and normalized volumes as per the below formula:

1. normalization factor = total volume (Intensity) reference lane/total lane stain-free volume (Intensity) of each lane.
2. normalized volume = normalization factor × volume (Intensity)

### 2.8. Biochemical Assay

L-lactate and glucose levels in blood and tissues were measured using a Lactate/Glucose Colorimetric Assay Kit (Abcam, Cambridge, MA, USA). Tissue redox markers of the NADH/NAD^+^ and NADPH/NADP^+^ ratio were analyzed by oxidized lactate with lactate dehydrogenase to generate a product that interacts with a probe to produce a color at λ_max_ = 450 nm (ab65331) using Abcam quantitation kits.

### 2.9. Microbiological Assay for Folate Levels

The *L. casei* assay was performed according to a previously described protocol [30]. Different concentrations of folate standard solution/quantitative tissue homogeneous solution were prepared, of which 100 µL were transferred into a 96-well microtiter plate, then pulsed with 10 µL diluted bacterial solution before incubating at 37 °C for 24 h. The plate was determined at an optical density (OD) of 590 nm.

### 2.10. Statistical Analysis

All statistical analyses were performed using SAS, version 9.4 (SAS Institute, Cary, NC, USA) and SPSS Statistics, version 14 (SPSS Inc., Chicago, IL, USA). The continuous variables were compared using one-way ANOVA (analysis of covariance) followed by Scheffe’s post hoc test. Chi-squared test was used for categorial variables. *p* for interaction of drug and dietary treatment on lactate metabolic parameters was tested using two-way ANOVA analysis. The significance level was set at * *p* < 0.05 for all analyses.

## 3. Results

### 3.1. FD Effect on Lactate Production and Metastatic Potential of LC Growing at Tumor Malignancy Stages

**Figure 1:**
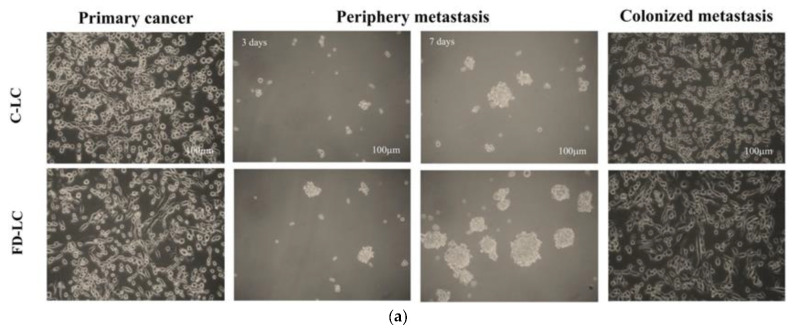

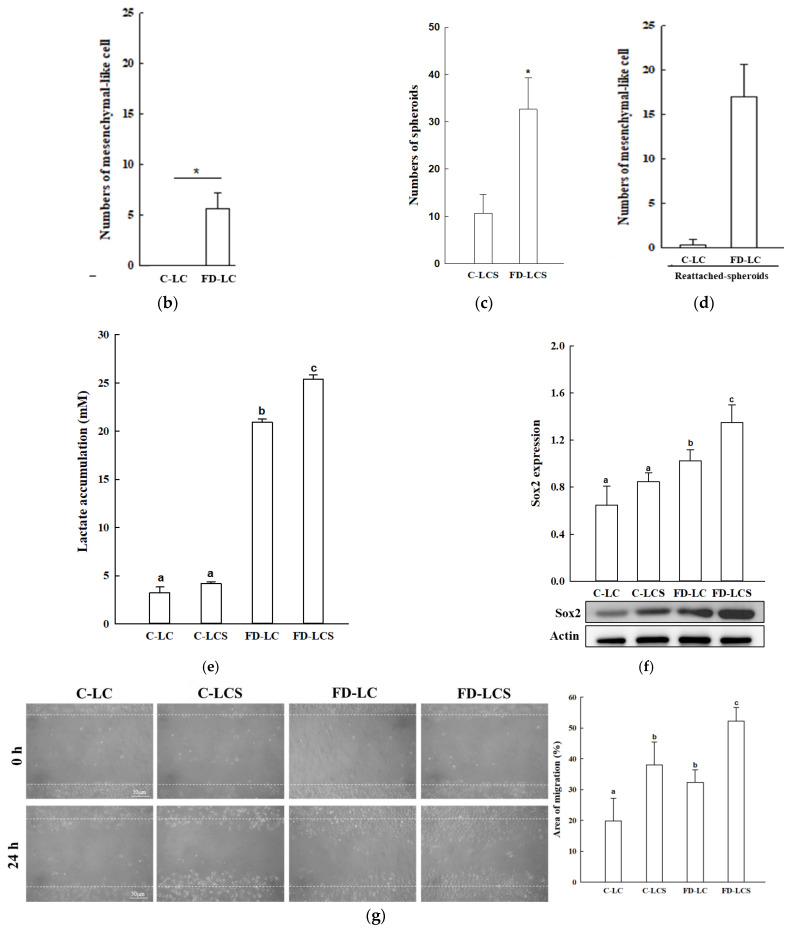

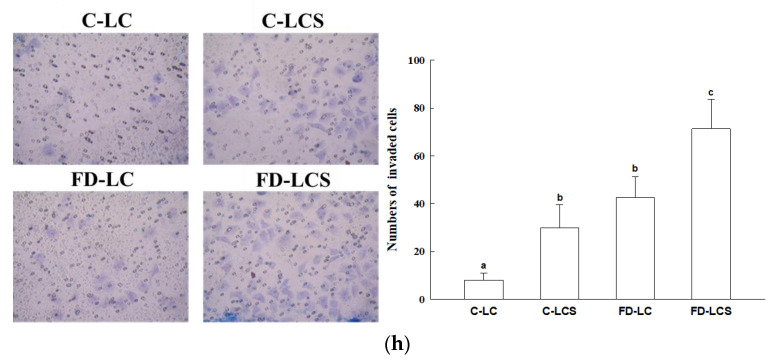
FD effect on lactate production and metastatic potential of LC growing at tumor malignancy stages. (**a**) Representative image of LCs growing in the control (C) and low folate-deficient (FD) medium at various stages of cancer malignancy. The culture protocols and treatments were detailed in the Methods section. Numbers of epithelial mesenchymal transition (ME) cells and oncospheroids growing at (**b**) primary cancers, (**c**) periphery metastases, and (**d**) recolonized metastases stages were quantified for the control and FD cells. (**e**) Lactate production, (**f**) expression of stemness marker SOX2, (**g**) migration, and (**h**) invasion of the control and FD-exposed cells growing at various malignancy stages. Data are expressed as the mean ± SD (n = 6 in each group). Statistical significance was analyzed by Student’s *t*-test (**b**–**d**) or by one-way analysis of variance, followed by Duncan’s multiple range test (**e**–**h**). Values without a common letter differed at *p* < 0.05. * indicates the comparison between the control group and FD groups by Student’s *t*-test at *p* < 0.05.

The causal effect of FD exposure on lactate production and metastatic potential of LC was investigated. To recapitulate tumor pathological course during cancer malignancy, LCs were cultivated on a monolayer growing as the primary tumors and cultured in control and FD medium for 4 days. The primary LCs were passaged into low-adherent plates for anchorage-independent oncospheroids’ growth for 7–9 days, designated as LCSs, to mimic aggressive tumors in periphery metastasis. These oncospheroids were then re-cultured into monolayers as the distant metastatic colonized tumors stage (Figure 1a). Compared with the LC cultivated in the control medium (C-LC), FD-LC induced the epithelial mesenchymal transition (EMT) phenotype, evident by significantly increased numbers of mesenchymal elongated cells (Figure 1a,b). Under anchorage-independent growth, FD-LC vs. C-LC cells formed larger oncospheroids (FD-LCSs) with 3-fold increased numbers of oncospheroids (Figure 1a,c). FD-LCS cells re-established EMT phenotype with 3-fold increased numbers of adherent cells (Figure 1a,d). As lactate release was measured under various LC growing stages, both C-LC and C-LCS had slight lactate production whereas FD-LC and FD-LDS displayed a significant 4–5-fold increase in lactate levels compared with their counterpart controls, with the highest lactate production by FD-LDS (Figure 1e). Expression level of the stemness marker, Sox2, as the metastatic index, was the highest in FD-LDS followed by FD-LD, whereas C-LC and C-LCS expressed significantly lower Sox2 levels compared with the FD counterparts (Figure 1f). FD-LCS displayed the highest metastatic potential as compared with the FD-LC and the control counterparts, evident by significant increased migration (Figure 1g) and invasive LC numbers (Figure 1h).

### 3.2. Effect of Dietary FD Intervention on Cancer Metastasis Efficiency and Lactate Metabolic Disorders in the LC-Implanted Mouse Model

**Figure 2:**
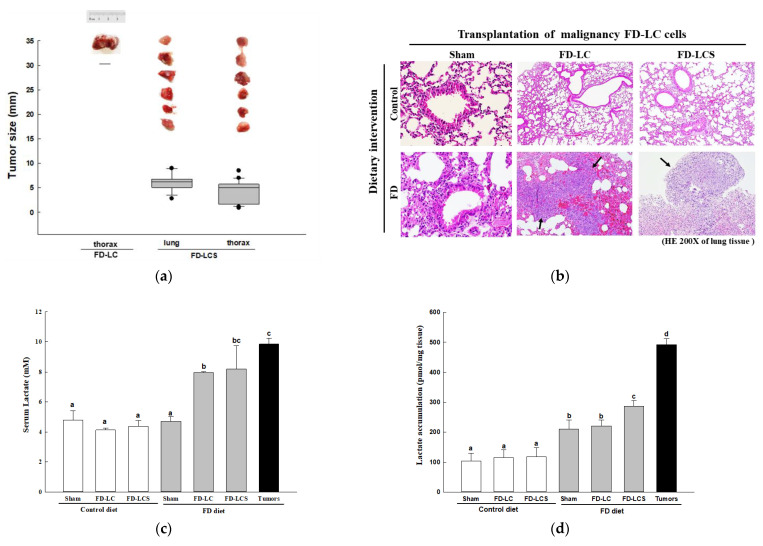

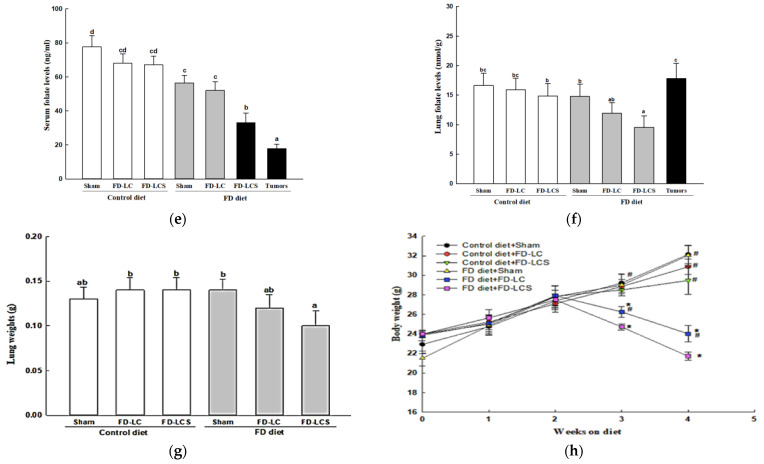
Effect of dietary FD intervention on cancer metastasis efficiency and lactate metabolic disorders in the LC-implanted mouse model. The C57BL/6 mice fed with the control (C) and folate-deficient (FD) diet were intrapleurally transplanted with LCs cultivated at various malignancy phases. The animal study protocols were detailed in the Methods section. (**a**) Representative images of metastatic lung and thorax tumors. (**b**) Hematoxylin and eosin staining of lung/tumors tissues in the control and FD mice receiving various transplants. The solid arrow indicates invasive tumors in the FD-LCS mice and pre-cancerous pathological lesions in the FD-LC mice. Serum, lungs, and tumor lactate (**c**,**d**) and folate (**e**,**f**) of the experimental mice groups. (**g**) Lung mass and (**h**) body weight of the experimental mice. Data are expressed as the mean ± SD (n = 4–8 as the respective indications). Statistical significance was analyzed using one-way analysis of variance, followed by Duncan’s multiple range test. Values without a common letter differed at *p* < 0.05. * indicates the comparison between the control group and FD groups. ^#^ indicates the comparison with the FD diet plus FD-LCS group. All statistics were performed through Student’s *t*-test at *p* < 0.05.

As C57BL/6 mice were pre-fed with the control and FD diets following intrapleural injection with in vitro control and FD-exposed LCs at various cancer malignant stages, only the FD-LCS transplants that produced high lactate (Figure 1e) could colonize the lung and thoracic tissues of the FD mice with a 100% tumorigenicity (Table 1). The FD-LCS-colonized tumors included 12 metastatic lung carcinomas and 22 metastatic sarcomas in the pectoral muscle of the thorax. Among these tumors, 92% of lung carcinomas and 55% of sarcomas proliferated into large tumors (tumor size > 5 mm) (Figure 2a). Histological examination on lung tissues of the experimental mice confirmed the invasive and proliferative lung tumors of the FD-LCS-implanted FD mice (Figure 2b). The FD-LC transplants with reduced lactate-producing capability (Figure 1e) only colonized one FD mouse to develop a large metastatic sarcoma in the thorax (Table 1 and Figure 2a) and induced lung pre-cancerous lesions despite the absence of lung tumors (Figure 2b). Both the FD-LCS and FD-LC transplants, however, did not develop metastatic cancers in the control-diet-fed mice, as evidenced by a 0% tumorigenicity rate (Table 1 and Figure 2a,b). Even for the C-LCS transplants that displayed migration and invasive capability yet produced little lactate (Figure 1e,h), they did not colonize any metastatic cancer in the FD mice (Table 1). As compared with the control and FD sham groups, the LCS-implanted FD mice developed a high-lactate and folate-deficient TME signified by 2-fold increased vascular lactate (Figure 2c), 1.5-fold increased lung lactate (Figure 2d), and decreased serum (Figure 2e) as well as lung folate (Figure 2f) compared with the other counterparts (*p* < 0.05). The metastatic lung tumors of the FD-LCS mice displayed the similarly high serum and lung lactate signature with their paired lung tissues (Figure 2c,d). Notably, metastatic lung tumors of the FD-LCS mice accumulated 2-fold higher folate levels than their tumor-adjacent lung tissues (Figure 2f), despite a 30–40% folate reduction in the paired lung tissues (Figure 2f) and serum samples of the FD-LCS mice (Figure 2e) compared with those respective folate pools of the FD sham and control counterparts. The results suggested folate redistribution in the TME of FD mice when harboring a heavy tumor load. Overall, the high lactate coupled with the FD-deprived (HL/FD) TME of the FD/LCS mice coincided with a systematic bioenergetic deficit, evident by a 20% lung tissue depletion (Figure 2g) and severe weight loss (Figure 2h), which resembled the symptoms of cancer cachexia in malnourished cancer patients with late-stage metastatic tumors and/or after anti-cancer chemotherapy.

### 3.3. Dietary FD and LC Invasion Modified Lactate Metabolic Targets in Lung and/or Metastatic Tumors of the Experimental Mice

**Figure 3:**
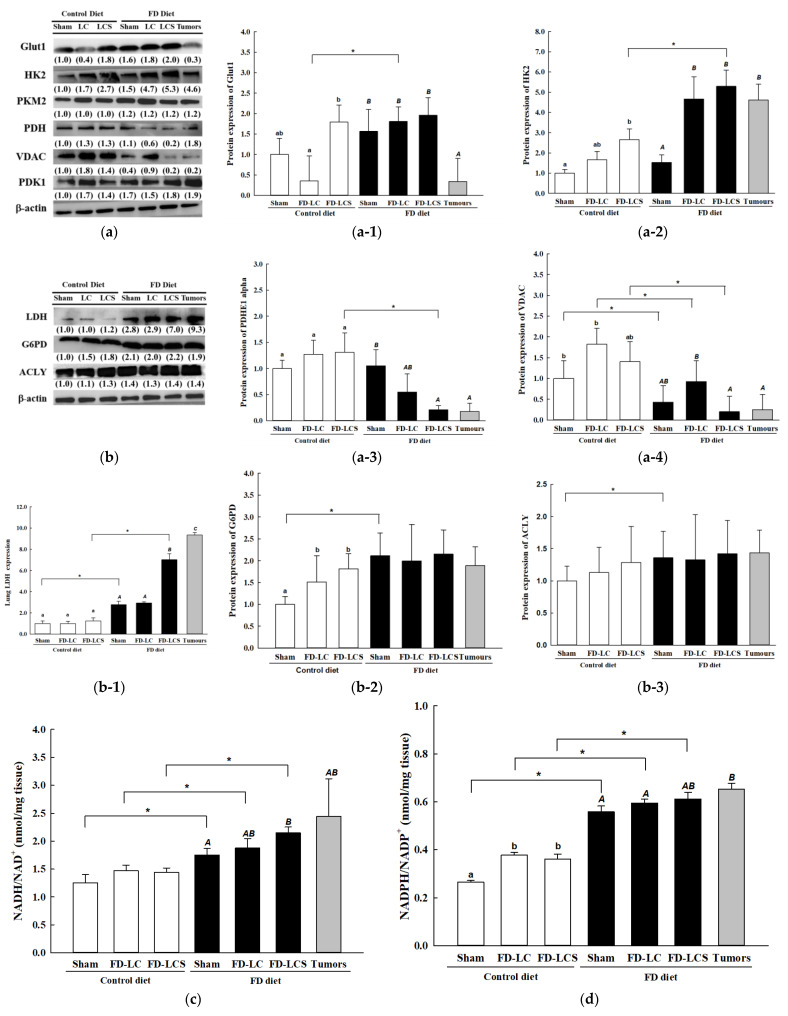
Dietary FD and LC invasion modified lactate metabolic targets in lung and/or metastatic tumors of the experimental mice. Representative gel image of oncotargets expressions (**a**,**b**) and lungs/paired tumors’ expression for (**a-1**) glucose transporter 1 (GLUT1); (**a-2**) hexokinase II (HK2); (**a-3**) pyruvate dehydrogenase E1 (PDHE1); (**a-4**) mitochondrial voltage-dependent anion channel protein (VDAC); (**b-1**) lactate dehydrogenase (LDH); (**b-2**) glucose-6-phosphate dehydrogenase (G6PD); (**b-3**) ATP-dependent citrate lyase (ACLY) of the control and FD mice with sham and LC transplantation were quantified by Western blot analysis. The reductive equivalents ratios as (**c**) NADH/NAD+ and (**d**) NADPH/NADP+ were determined as described in the Methods section. Values are expressed as mean ± standard deviation (n = 4–8 from each group). Statistical significance was analyzed using one-way analysis of variance, followed by Duncan’s multiple range test within each dietary treatment group. Values compare within Control diet with lowercase letters and FD diet with uppercase lettersat *p* < 0.05. * indicates the comparison between the control group and FD groups by Student’s *t*-test at *p* < 0.05.

**Figure 4:**
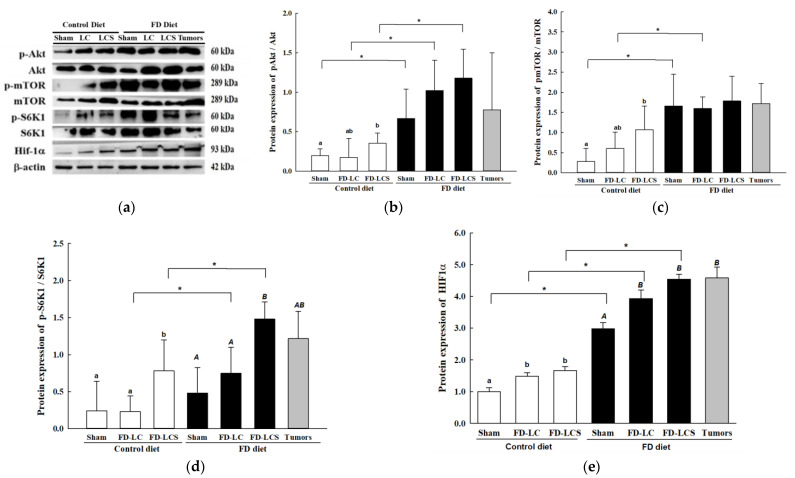
Dietary FD and LC invasion activated the metabolic stress–mTOR-signaling pathways. Representative gel image (**a**) and total and/or phosphorus–protein expressions levels in (**b**) Akt, (**c**) mTORC1, (**d**) S6K1, and (**e**) HIF1α of the control and FD mice in the absence (sham) and presence of LC transplantation were quantified by Western blot analysis. All the phosphorylated proteins were normalized by their respective unphosphorylated form. Statistical significance was analyzed using one-way analysis of variance, followed by Duncan’s multiple range test within each dietary treatment group. Values compare within Control diet with lowercase letters and FD diet with uppercase at *p* < 0.05. * indicates the comparison between the control group and FD groups through Student’s *t*-test at *p* < 0.05.

To unveil the decisive factors involved in dietary FD- and invaded LC-induced lactate metabolic disorder, expressions of rate-limiting enzymes in aerobic glycolysis and mt OXPHOS in lung/tumors of the control and FD mice were assayed by Western blot. As shown in Figure 3, the FD/LCS mice, relative to the FD sham mice and/or the FD-LC mice, had significantly increased lung expressions of HK2 (Figure 3(a-2)), and reduced expressions of PDHE1 (Figure 3(a-3)) and mt voltage-dependent anion channel protein (VDAC) (Figure 3(a-4)). Compared with the FD sham mice, the enhanced glycolysis and suppressed mt OXPHOS enzyme expressions of the FD-LDS mice were coupled with increased lung expression of LDH for lactate over-production (Figure 3(b-1)). The metastatic lung tumors of the FD/LDS mice displayed a similar alteration of expression profile in lactate-metabolizing enzymes with their paired lung tissues (Figure 3(a-2–a-4),(b-1)), except for GLUT1. The FD diet alone (FD sham) or in combination with LC and LCS transplants did not significantly alter lung expression of GLUT1, yet down-regulated expression of tumors’ GLUT1 (Figure 3(a-1)).

As compared to mice fed with the control diet alone (the C sham group), dietary FD per se (FD sham group) significantly suppressed lungs’ mt VADC (Figure 3(a-4)) and promoted LDH expression (Figure 3(b-1)) without affecting expressions of the other tested glycolytic enzymes (Figure 3a,b). Expression of rate-limiting enzymes for anabolic metabolism such as G6PD (Figure 3(b-2)) for the pentose phosphate pathway (PPP) and ACLY (Figure 3(b-3)) for fatty acid synthesis was both significantly up-regulated in the FD sham group vs. the C sham group. In parallel, the redox equivalents as ratios of NADH/NAD+ (Figure 3c) and NADPH/NADP+ (Figure 3d) to support lungs’ anabolism were significantly increased in the FD vs. control sham groups. The enhanced lactate-associated anabolic bioenergetic metabolism in lungs of the FD sham mice remained up-regulated in lungs and also in metastatic tumors of the LCS mice (Figure 3b–d). For the control mice, neither LC nor LCS transplantation altered expressions of LDH (Figure 3(b-1)), anabolic enzymes (Figure 3(b-2,b-3)), and redox status of NADH/NAD+ (Figure 3c), despite the fact that expressions of glycolytic enzyme HK2 (Figure 3(a-2)), mt VDAC (Figure 3(a-4)), and NADPH/NADP+ ratio (Figure 3d) were in part increased by LC or LCS transplants.

### 3.4. Dietary FD and LC Invasion Activated the Metabolic Stress–mTOR-Signaling Pathways

Next, we explored whether and how the dietary FD and implanted LC may impact on mTOR-signaling pathways, the master metabolic regulator of glycolytic and mt OXPHOS metabolism. As shown in Figure 4, the FD sham mice relative to the control sham group displayed significantly increased lung p-Akt levels, the upstream regulator of mTOR signaling. This FD-activated signal was further significantly enhanced by FD-LCS transplants in lungs and paired tumor tissues, evidenced by increased p-Akt level expression as compared to those of the FD sham group (Figure 4a,b). Correspondingly, FD activation of mTOR-signaling pathways was apparent by significantly increased expression of pmTOR (Figure 4a,c) and its two downstream targets of p-S6K1 (Figure 4a,d) and HIF1α (Figure 4a,e) in the FD sham mice compared with the control sham mice. LC and LCS transplantation of the FD mice maintained the FD-activated pmTOR (Figure 4b) and HIF1α levels (Figure 4d), and significantly enhanced p-S6K1 expressions (Figure 4c). Paired tumors from the FD/LCS mice displayed a similar activated mTOR-signaling profile with their paired lung tissues (Figure 4a–e). The impact of LC transplantation on mTORC1 activation of the FD mice at expression levels of pmTOR, p-S6K1, and HIF1α was significantly higher in the FD mice than in the control counterparts (Figure 4a–e), suggesting the synergistic effect of FD diet and LC invasion, in particular for FD-LCS, on mTOR metabolic signaling transduction.

### 3.5. Efficiency of Anti-Neoplastic Drug Treatments Rapamycin (Rap) and Metformin (Met) on FD-LCS-Induced LC Metastasis

**Figure 5:**
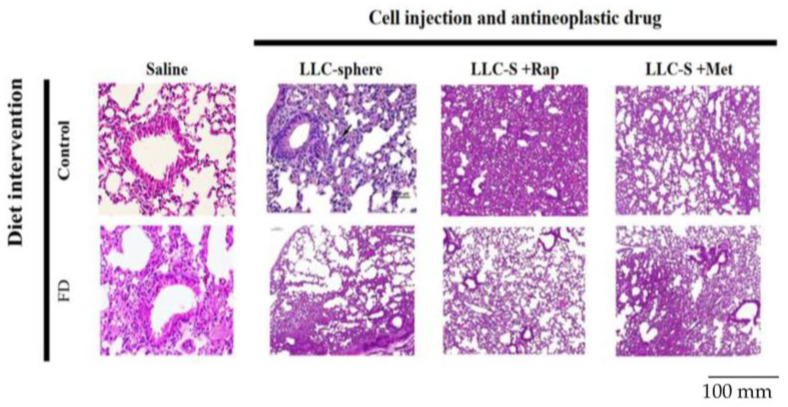
Treatment efficiency of anti-neoplastic drugs rapamycin (Rap) and metformin (Met) on the FD-LCS-induced LC metastasis. The C57BL/6 mice were fed with the control (C) and folate-deficient (FD) diet for 14 days and were intrapleurally implanted with saline (sham group) and the FD-LCSs. Drug treatment of rapamycin (Rap: 0.21 mM) and metformin (Met: 1.5 mM) at dose of 200 mg/mL/day was conducted by intraperitoneal injection to the control and FD mice one week prior to LCS transplantation. The experimental protocols were detailed in the Methods section. (Hematoxylin and eosin staining of the lung/tumor tissues in the control and FD mice receiving FD-LCS transplants and drugs treatment. Black arrow bars indicate cancerous lesions with chronic inflammation and lymphocyte aggregation.

We evaluated the drug effects of rapamycin (Rap), an mTORC1-signaling blocker, and the anti-diabetic drug metformin (Met) on FD/LCS-potentiated cancer metastasis. As shown in Table 2, the FD-LCS transplantation to the FD mice resulted in a 100% metastatic tumor colonization rate (8/8) with LC type of lung carcinoma and sarcomas in the pectoral muscle of the thorax, similar to the FD-induced tumorigenicity rate in our prior findings in Table 1 and Figure 2. Pre-treatment of the FD/LCS-implanted mice with Rap and Met drugs essentially prevented LC metastasis in lung and thorax tissues, resulting in a 0% metastatic tumor colonization rate (0/6). Neither Rap nor Met pre-treatment of the LCS-implanted mice fed with the control diet affected the 0% metastatic tumor colonization rate found in the control/LCS-implanted mice. Histological examination of lung tissues of these experimental mice revealed pathological lesions with chronic inflammation and lymphocyte aggregation in tumor-associated lungs of the FD/LCS-implanted mice compared with lungs of the control and FD sham mice (saline injection) (Figure 5). Rap and Met treatment of the LCS-transplanted control and FD mice did not result in any pre-cancerous lesions in lungs. The data suggested that Rap and Met treatment inhibited LCS metastases in the FD mice with no cancer effect on the control mice.

### 3.6. Druggable Protein Targets of Rap and Met Treatment in FD/LCS-Activated mTORC1 Signaling and Lactate Metabolic Disorder

**Figure 6:**
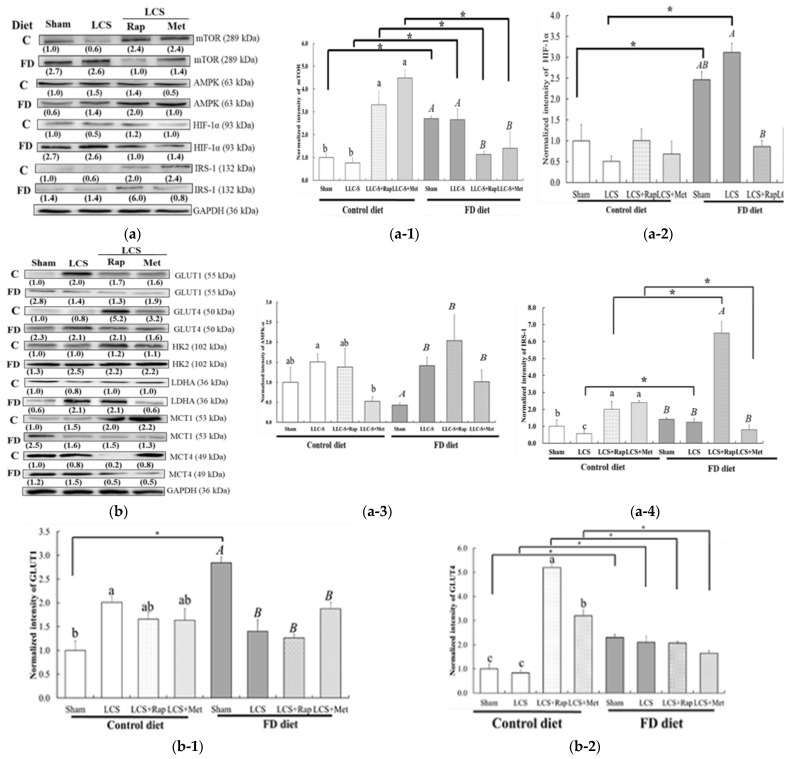

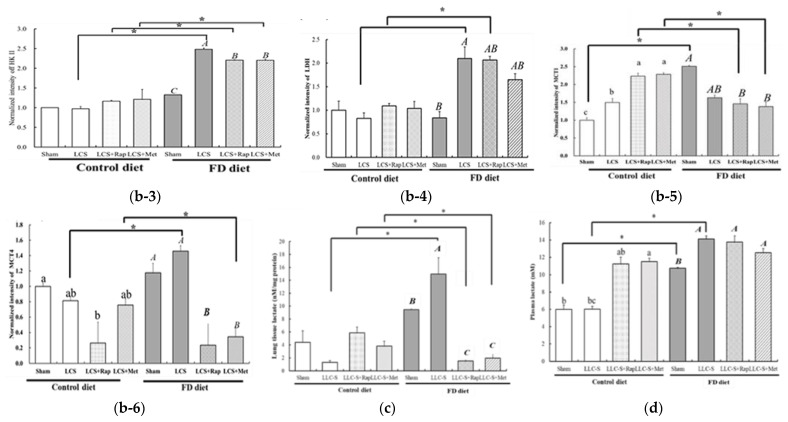
Druggable protein targets of Rap and Met treatment in FD/LCS-activated mTORC1 signaling and lactate metabolic disorder. Representative gel images (**a**,**b**) and lung protein expressions including (**a-1**) mTORC1 (**a-1**); HIF1α (**a-2**); AMPK (**a-3**); IRS (**a-4**); GLUT1 (**b-1**); GLUT4 (**b-2**); Hk2 (**b-3**); LDHA (**b-4**); MCT1 (**b-5**); and MCT4 (**b-6**) were analyzed by Western blotting. (**c**) Lung and (**d**) blood lactate of the experimental mice were evaluated according to the protocols detailed in the Methods section. Values are expressed as the mean ± SD (n = 3–6 in each group). Statistical significance was analyzed using one-way analysis of variance, followed by Duncan’s multiple range test. Values without a common letter differed at *p* < 0.05. * indicates a comparison between the control and FD groups by Student’s *t*-test at *p* < 0.05.

Towards the end, we deciphered the druggable targets associated with their anti-FD/LCS-induced cancer metastasis. As shown in Figure 6a, Rap treatment abolished FD/LCS-activated mTOR signaling by decreasing the expression of mTORC1 (Figure 6(a-1)) and HIF1α (Figure 6(a-2)), retaining LCS-induced 2-fold increased expression of AMP-dependent protein kinase (AMPK) expression (Figure 6(a-3)), and promoting 5-fold increased expression of insulin receptor substrate (IRS) expression (Figure 6(a-4)) as compared to those in the FD sham and FD/LCS-implanted mice. Rap blockage of FD/LCS-activated mTOR/HIF1α signaling coincided with moderate yet significant inhibition of FD/LCS-induced lung expression of HK2 (Figure 6(b-3)) and a non-significant reduced expression of LDHA (Figure 6(b-4)). In particular, a significant 50% reduced expression of FD/LCS-activated MCT4 (Figure 6(b-6)), a monocarboxylate transporter (MCT4) for lactate exportation, was evident after Rap treatment as compared to their untreated FD counterpart. Rap treatment in mice did not significantly alter FD/LCS-inhibited expression of GLUT1 (Figure 6(b-1)) and GLUT4 (Figure 6(b-2)) and had little impact on FD/LCS-promoted MCT1 (Figure 6(b-5)), the lactate importer (Figure 6(b-4)), as compared with their untreated counterpart.

Similar to Rap’s inhibitory effect on mTOR/HIF1α/AMPK signaling, Met treatment abrogated FD/LCS-promoted expression of mTORC1 (Figure 6(a-1)) and HIF1α (Figure 6(a-2)), and sustained FD/LCS-promoted AMPK expression (Figure 6(a-3)) compared with their untreated FD and control counterparts. The striking differential effect of Met treatment from Rap was observed for the IRS target (Figure 6(a-4)), in that Met treatment inhibited LCS-promoted IRS expression, whereas Rap treatment displayed the opposite effect to further the up-regulation of LCS-induced IRS expressions when compared with the control-drug-treated counterparts.

Similar to Rap’s modulating effect on the downstream targets of mTOR/ HIF1α/AMPK signaling, Met blockage of the FD/LCS-activated mTOR/HIF1α signaling coincided with significant inhibition of FD/LCS-induced lung expression of HK2 (Figure 6(b-3)) and non-significant reduced LDH expression (Figure 6(b-4)). In particular, Met treatment induced a significant 50% reduced expression of FD/LCS-activated MCT4 (Figure 6(b-6)) in lungs as compared to their FD sham and untreated FD counterparts. Met treatment did not alter FD/LCS-inhibited expression of GLUT1 (Figure 6(b-1)) and GLUT4 (Figure 6(b-2)) and had little impact on FD/LCS-promoted MCT1 (Figure 6(b-5)), the lactate importer, compared with their untreated counterpart.

Reversion of FD/LCS-reprogramming lung mTOR/HIF1α/AMPK/IRS signaling and lactate mobilization target expression by Rap and Met subsequently resulted in a significant 7-fold decrease in lung lactate levels compared with their control and untreated counterparts (Figure 6c). Plasma lactate of the FD/LCS mice were little modulated by Rap and Met treatment compared with their drug-untreated counterpart (Figure 6d). The dietary folate intervention significantly interacted with metastatic LCSs (*p* for interaction = 0.024) and two drug treatments (*p* for interaction = 0.001) to reduce lung lactate.

It is noticeable that Rap and Met treatment of the control mice simultaneously induced 2-fold increased levels of plasma lactate with no effect on the control lungs’ lactate, compared with those of their control sham and control drug-untreated counterparts. The data suggested an off-lung target effect of the drugs on the lactate metabolism of other organs in the control mice. Despite the lack of drug effect on lung lactate levels of the control, both Rap and Met treatment did promote control lungs’ expression of mTORC1 and IRS (Figure 6(a-1–a-4) and increased expression of GLUT4 and MCT1 for glucose/lactate uptake (Figure 6b), without affecting HIF1α/AMPK expression by Rap and by reducing AMPK expression by Met (Figure 6(a-1–a-4)). No drug impact on expression of enzymes in aerobic lactate production (HK2 and LDH) and lactate exporter MCT4 of the control mice was found as compared to the drug-untreated control counterparts.

## 4. Discussion

To our knowledge, this is the first study to unveil the lactate metabolic influence of dietary FD to promote lung carcinoma metastases. Implantation of FD-induced high-lactate-producing LCSs to the FD but not control mice resulted in a specific hyperlactatemic TME in the vascular system and lungs to guarantee 100% LCS metastases. This newly identified lactate metabolite biomarker of folate deficiency as a function of LC malignancy is concurrent with numerous clinical studies reporting hyperlactatemia being the diagnostic and prognostic predictor for tumor metastasis and recurrence in lung and other cancers [19,20,21,22]. How folate malnutrition elicits metabolic disorder of hyperlactatemia for LC malignancy remains elusive. In the content of the lactate biosynthesis pathways, over-expression of hexokinase 2 (HK2) and lactate dehydrogenase A (LDHA), the committed steps to regulate magnitude and direction of glucose flux for lactate production, are required for tumor initiation, maintenance, and metastatic cancer spread [31,32]. Inactivation of pyruvate dehydrogenase (PDH) by pyruvate kinase (PK) will block pyruvate entrance into the Krebs cycle to reduce mt OXPHOS and spare glucose carbon for lactate production [33,34]. Several metabolic flux studies revealed that ^13^C-lactate/glucose fueled aerobic glycolysis and the TCA cycle to sustain the anaplerotic biosynthetic need for the metastatic spread of human lung cancers [25,35,36]. In line with the malignant phenotype of lactate-metabolizing signatures, our data demonstrated that the FD/LCS-metastatic mice reprogrammed lung/metastatic tumors’ fuel metabolism from mt OXPHOS, by suppressed expression of PDH and mt VDAC, to accelerated aerobic glycolysis, by increased expression of HK2, LDHA, and PKM2, for lactate over-production. The data signify the FD/LCS-induced Warburg effect in metastatic tumors/lung tissues. In line with the lactate-acidified TME to shape the metastatic behavior of cancers [15,16,17,18], our data demonstrated that FD exposure of lung adenomas resulted in lactate secretion to acidify the TME and enhanced migration and invasive potentials to support anchorage-independent oncospheroid growth at the peripheral cancer metastatic stage. Even under FD-induced bioenergetics deficit from mt oxidative damage and dysfunctional mt OXPHOS [12,13,14], the FD-promoted hyperlactatemic TME of the vascular system and lungs confer the metastatic advantage of malignant cancer to acquire the alternative lactate fuel for survival and anchorage-independent growth in peripheral conditions, resulting in a 100% metastatic rate.

Of the most significant findings, our data demonstrated the molecular regulation of FD/LCS-reprogramming lactate metabolic disorder and LC malignancy through activation of the mTORC1/HIF-1α-signaling pathway, the energy sensor and master regulator of cell fuel metabolism [37]. In the FD sham and FD/LCS mice compared with the control counterparts, activation of lungs’ mTORC1 signaling cascade was evident with up-regulation of the upstream mediator, phosphorylated Akt (p-Akt), which activates phosphorylation of mTORC1 (mechanistic target of rapamycin) kinase and its downstream effector, the ribosomal p70-S6 kinase (pS6k), to stimulate ribosomal activity for protein biosynthesis and the signaling target of hypoxia-inducing factor (HIF-1α) [38]. HIF-1α is a well-characterized powerful transcriptional factor promoting aerobic glycolysis by up-regulation of the expression of GLUT1, HKII, and LDHA for increased glucose uptake and accelerated aerobic glycolysis for lactate production [39]. By the use of lactate as the anabolic fuel source, activation of mTORC1/ HIF-1α in a metastatic-cancers-driven TME has been demonstrated to switch cell lactate metabolism toward increased production of protein, lipids, and nucleotides for tumor biomass expansion and cancer proliferation [40]. Activation of mTORC1 stimulates de novo purine synthesis through control of the mt tetrahydrofolate cycle targeting methylenetetrahydrofolate dehydrogenase 2 (MTHFD2) in serine-driven one-carbon metabolism which raises cellular NADPH/NADP+ and GSH/GSSG ratios to confer anabolic metabolic advantages for cancer metastasis [41,42]. In line with the mTORC1-signaling lactate anabolic metabolism, the FD/LCS-activated mTORC1/HIF-1α signaling cascade coincided with increased lung/tumor expression of rate-limiting enzymes for anabolic metabolism such as G6PDH which drives to the pentose phosphate pathway for nucleotide synthesis, ACLY for fatty acid synthesis, and empowers redox potential by increased ratio of NADPH/NADP+ and NADH/NAD+ in paired lung tumors. Furthermore, activation of mTORC1/HIF-1α signaling regulates lactate/proton symporters of MCT1 for lactate import and MCT-4 for lactate export, which mobilize lactate fuel transfer between stromal and cancer cells to sustain energy supply for cancer malignancies [43]. High lactate primes human fibroblasts to promote mTOR/HIF-1α-mediated lactate production and stromal lung expressions of MCT4 to export lactate for feeding tumors’ energy demands [44]. Knockdown of MCT4 inhibited invasiveness of human lung cancer cells [45]. Over-expression of GLUT1 and MCT4 in lung adenocarcinomas was associated with a poor disease-specific survival [46]. Increased MCT4 expression in cancer cells and stromal cells correlates with worse prognosis across many cancer types including lung cancer [47]. In the present study, blockage of the FD/LCS-activated mTOR/HIF1α signaling by the mTORC1 inhibitor, Rap, abrogated FD/LCS-promoted lungs’ glycolytic lactate production and exportation in lungs by significant inhibition of HK2 and MCT4 expressions to impede lactate supply for metastatic cancer use, and subsequently prevented LCS metastases development. Collectively, our findings identified the Rap druggable biomarkers of mTORC1/HIF-1α signalers and MCT4 lactate exporter as the most plausible anti-cancer therapeutic targets for reversing nutritional FD-potentiated lactate disorders and LCS metastases.

**Figure 7:**
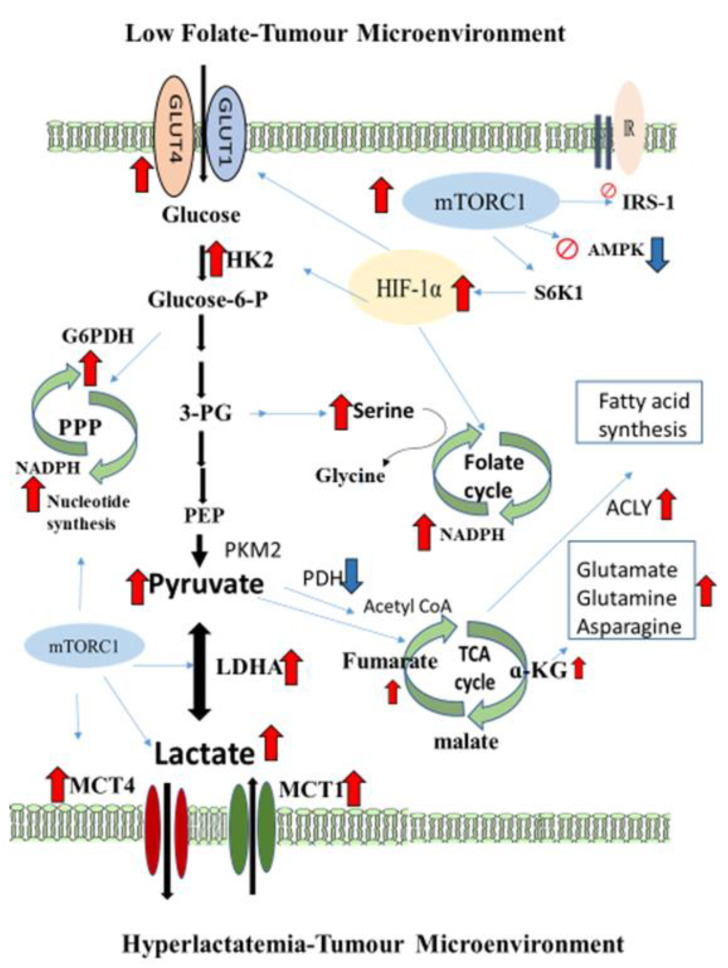
Integrative FD-reprogramming lactate metabolite signatures and mTOR-signaling-mediated druggable targets in FD/LCS-promoted LC metastatic tumor microenvironment. Red bar arrows indicate up-regulation. Blue bar arrows indicate down-regulation.

More clinically significant, we demonstrate that the FD/LCS-driven lactate metabolic vulnerability for LC metastases can be therapeutically targeted by Met, the conventional anti-diabetic medicine, which has been under extensive pre-clinical investigation and clinical trials for its therapeutic effect of lung cancers [48,49] Resembling the Rap effect, Met treatment inhibited FD/LCS-activated mTORC1 signaling by suppressed expression of mTORC1/HIF-1α signalers, and decreased HKII and the MCT4 lactate exporter to result in Met normalizing lungs’ lactate levels. The molecular signaling targets of Met-treated FD/LCS mice are in line with the previous studies reporting on Met treatment of lung non-small cell lung cancer [50], adenocarcinomas [51], large-cell lung cancers [52], and of Balb/c nude mice implanted with A549 adenocarcinoma cells [53]. Numerous pre-clinical and retrospective clinical studies reported that Met treatment exerts its anti-neoplastic effects in sensitizing diabetic NSCLC patients to cytotoxic drug therapy on activation of the AMP-activated kinase pathway, an early growth signal transduction to counteract mTOR pathway activation in lipid and protein metabolism and in particular to promote apoptotic cancer death [48,49]. In the present study, AMPK expression was the dual target for FD/LCS and drug treatment in that Met/Rap treatment of the FD/LCS mice sustained the FD/LCS-activated AMPK expression with deactivation of mTOR/HIF-1α signaling and its downstream protein expressions. The integrative FD-reprogramming lactate metabolite signatures and mTOR/AMPK/HIF-1α-signaling-mediated druggable targets in the FD/LCS-promoted LC metastatic tumor microenvironment are illustrated in Figure 7. Activation of the AMPK/mTOR/HIF-1α loop-signaling pathway has been identified as the key anti-cancer mechanism to mediate metabolic check-points such as p53 and p21, and induced cell death modes in apoptosis and autophagy upon these cytotoxic drug treatments [40]. A deeper mechanistic insight into AMPK/mTOR-signaling cytotoxic targets upon Met/Rap treatment of FD/LCS mice warrants further studies.

It should be noted that Rap/Met treatment exerted little impact on FD/LCS-induced hyperlactatemia yet elicited a two-fold increase in plasma lactate with no lung lactate effect in the control mice. The uncoupling drug effects on lungs and vascular lactate levels of the control and FD/LCS-implanted mice could not be simply attributed to drug-induced lung expression of mTORC1/IRS1/GLUT4/MCT1 for glucose/lactate shuttling between TME compartments of the controls and FD mice. Upon a diverse set of environmental stimuli on metabolic complexity, the FD diet and LCS invasions significantly interacted to complicate drug treated efficiency not only on lung targets, as disclosed in the present study, but also likely on several plausible off-targets of the host in fuel-utilizing organisms such as liver, muscle, and brain which encompass inherent genomic instability and phenotypic plasticity, heterogeneous bioenergetics demands, and differential metabolic mTORC1 signaling regulation in glucose and lactate utilization and mobilization [40]. In human cancer patients, FD-induced low serum folate has been associated with increased risks of tumor late-stage malignancy and poor prognosis rate [3,4]. The FD-diet-generated lactate metabolic susceptibility of the host at the tumor invasive stage, a common clinical malnutrition that cancer patients with cancer cachexia and/or adjuvant chemotherapy frequently encounter and that may not have been diagnosed for targeted effective therapy, may allow cancers to rapidly metastatically evolve and/or give rise to drug-resistant subclones, leading to tumor relapse and therapy failure. Precision targets on the FD-diet- and LCS-induced druggable oncoproteins warrant further studies to provide effective intervention strategies to cope with therapeutic perturbation of cancer lactate metabolism.

## 5. Conclusions

In summary, the present in vivo and in vitro study delineates FD-induced and mTORC1/AMPK//HIF-1α-signaling-mediated lactate metabolism disorders in lung cancer metastasis. For the first time, we characterize mTORC1-metabolite sensing and signaling networks to mediate dietary-folate-malnutrition-induced cancer malignancy hallmarks and oncotargets of Rap and Met treatment in lactate metabolic disorders. Our pre-clinical studies unveil novel FD-reprogramming druggable targets on lactate metabolic biomarkers, which would facilitate future clinical investigation to open a new therapeutic window for optimizing nutrition and drug intervention in order to maximize prognosis of metastatic lung cancers.

## Figures and Tables

**Table 1:** Metastatic tumor burden, tumor incidence, colonization rate, and colonization target tissues of the experimental mice group.

	Control Diet				FD Diet				
	Sham	FD-LC	FD-LCS	C-LCS	Sham	FD-LC	FD-LCS		C-LCS
Colonization rate (%)	0/4 (0)	0/6 (0)	0/8 (0)	0/4 (0)	0/6(0)	1/8 (12.5) *	8/8 (100) ^†^		0/5 (0)
Tumor burden						Thorax	Lung	Thorax	
Total multiplicity, n	0	0	0	0	0	1	12	22	0
weight (g)	0	0	0	0	0	3.47	0.06 ± 0.04	0.27 ± 0.80	0
size (mm)	0	0	0	0	0	30.24	6.09 ± 1.68	4.00 ± 5.95	0
size > 5 mm, n	0	0	0	0	0	1	11	12	0

C57BL/6 mice were fed with folic acid deprivation or control diet and received intrapleural injection with the Lewis lung carcinoma cells (LC) or spheroids (LCS) cells (1 × 10^6^). C: control medium; C-LC and C-LCS cells both were cultivated with control-medium; FD: folic acid deprivation medium; FD-LC and FD-LCS cells both were cultivated with folate deprivation (FD)-medium. * *p* < 0.05 compared with control diet FD-LC group. † *p* < 0.05 compared with FD diet FD-LC group. Student’s *t*-test was used to analyze the statistical significance of differences.

**Table 2:** Summary of metastatic tumor burden including metastatic cancer colonization rate and sites in the experimental mice.

Dietary Intervention	FD-LCS Transplantation	Metastatic Tumor Colonization, Rate (%)	Metastatic Tumor Site	
			Lung (n/Total)	Thorax (n/Total)
	Sham	0/4 (0)	0	0
Control	LCS	0/6 (0)	0	0
	LCS + Rap	0/5 (0)	0	0
	LCS + Met	0/3 (0)	0	0
	Sham	0/6 (0)	0	0
FD	LCS	6/6 (100)	12/27	15/27
	LCS + Rap	0/6 (0)	0	0
	LCS + Met	0/6 (0)	0	0

Male C57BL/6 mice at 6 weeks of age were fed an amino-acid-defined folate-deficient diet (FD group) or a control diet (Control: +folate at 2 mg/kg FD diet) for 14 days. The FD and control mice were then intrapleurally implanted with saline (sham group) and FD-exposed lung carcinoma spheroids (LCSs) at density of 1 × 10^6^ cells. Drug treatment of rapamycin (Rap) and metformin (Met) at dose of 200 ug/kg body weight was conducted by intraperitoneal injection to the control and FD mice one week prior to LCS transplantation.

## Data Availability

Not applicable.

## Supplementary Materials

The following supporting information can be downloaded at: https://www.mdpi.com/article/10.3390/nu15061514/s1, Table S1: The composition of folic acid deficient diet.
