# Supplementary material for: Dietary Folate Deficiency Promotes Lactate Metabolic Disorders to Sensitize Lung Cancer Metastasis through MTOR-Signaling-Mediated Druggable Oncotargets
Source: Nutrients. 2023 Mar 21;15(6):1514. doi: 10.3390/nu15061514 (PMC10052195; doi:10.3390/nu15061514)
Supplement: Supplementary file 1 [file nutrients-15-01514-s001.zip › nutrients-2273353-supplementary.pdf]

Table S1. The composition of folic acid deficient diet.

Formula	g/Kg
Sucrose	357.1526
Corn Starch	150.0
Maltodextrin	160.0
Corn Oil	80.0
Cellulose	30.0
Mineral Mix, AIN-76 (170915)	35.0
Calcium Phosphate, dibasic	3.0
L-Alanine	3.5
L-Arginine HCl	12.1
L-Asparagine	6.0
L-Aspartic Acid	3.5
L-Cystine	3.5
L-Glutamic Acid	40.0
Glycine	23.3
L-Histidine HCl, monohydrate	4.5
L-Isoleucine	8.2
L-Leucine	11.1
L-Lysine HCl	18.0
L-Methionine	8.2
L-Phenylalanine	7.5
L-Proline	3.5
L-Serine	3.5
L-Threonine	8.2
L-Tryptophan	1.8
L-Tyrosine	5.0
L-Valine	8.2
Vitamin C, ascorbic acid, coated (97.5%)	1.02
Biotin	0.0004
Vitamin B 12 (0.1% in mannitol)	0.03
Choline Dihydrogen Citrate	3.5
Calcium Pantothenate	0.066
Inositol	0.11
Vitamin K3, menadione	0.05
Niacin	0.099
Pyridoxine HCl	0.022
Riboflavin	0.022
Thiamin (81%)	0.022
Vitamin E, DL-alpha tocopheryl acetate (500 IU/g)	0.242
Vitamin A Palmitate (500,000 IU/g)	0.0396
Vitamin D 3, cholecalciferol (500,000 IU/g)	0.0044
Ethoxyquin, antioxidant	0.02

Footnote: An amino acid defined diet (used to limit background folic acid) without folic acid added (Teklad Custom Diet, TD.00434). This diet does not contain succinylsulfathiazole, which is used in quite a few folic acid deficient diets. TD.110609 is a possible control that adds folic acid at 2 mg/kg diet.
